# Development of the Italian version of the High-Activity Arthroplasty Score (HAAS-I) following hip and knee total arthroplasty: cross-cultural adaptation, reliability, validity and sensitivity to change

**DOI:** 10.1186/s13018-018-0782-5

**Published:** 2018-04-11

**Authors:** Marco Monticone, Antonio Capone, Luca Frigau, Giuseppe Marongiu, Paola Abelli, Francesco Mola, Nicola Maffulli, Calogero Foti

**Affiliations:** 1 Department of Public Health, Clinical and Molecular Medicine, University of Cagliari, Cagliari, Italy; 2 Orthopaedic Unit, Department of Surgery, University of Cagliari, Cagliari, Italy; 3 Department Economics and Business Science, University of Cagliari, Cagliari, Italy; 4 Scientific Institute of Montescano, Clinical and Scientific Institutes Maugeri, Institute of Care and Research, Montescano, Pavia Italy; 5 Department of Musculoskeletal Disorders, University of Salerno School of Medicine and Surgery, Salerno, Italy; 6 Centre for Sports and Exercise Medicine, Barts and The London School of Medicine and Dentistry, Mile End Hospital, 275 Bancroft Road, London, UK; 7 Physical Medicine and Rehabilitation Unit, University of Rome “Tor Vergata”, Rome, Italy

**Keywords:** HAAS, Cross-cultural adaptation, Italian validation, Total hip arthroplasty, Total knee arthroplasty

## Abstract

**Background:**

The number of physically active individuals who develop knee and hip arthritis and who undergo arthroplasties of these joints ie ever increasing. It has become necessary to develop evaluation scales which address the specific issues raised by such individuals. The High Activity Arthroplasty Score is one such scales, originally developed in English.

**Methods:**

The HAAS-I was developed by means of forward-backward translation, a final review by an expert committee and a test of the pre-final version to establish its correspondence with the original English version. The psychometric testing included reliability by means of internal consistency (Cronbach’s alpha) and test–retest reliability (intraclass correlation coefficients) and construct validity by Pearson’s correlations with a pain intensity numerical rating scale (NRS), the Western Ontario and McMaster University index (WOMAC, for THA subjects), the Knee injury and Osteoarthritis Outcome Scale (KOOS; for TKA subjects) and the Short-Form 36 Health Survey (SF-36).

**Results:**

The questionnaire was administered to 67 subjects with THA and 61 with TKA and proved to be acceptable. The questionnaire showed good internal consistency (0.85 for THA and 0.91 for TKA) and a high level of test–retest reliability (ICC = 0.97 with 95% CI 0.95–0.98 for THA; ICC = 0.95 with 95% CI 0.92–0.98 for TKA). There was a moderate correlation between the HAAS-I and NRS (*r* = − 0.40), there was a high correlation between the HAAS-I and WOMAC (*r* = − 0.68) and there were moderate to high correlations between the HAAS-I and SF-36 subscales (*r* = 0.34 to 0.63) for THA. There was a moderate correlation between the HAAS-I and NRS (*r* = − 0.77); there was a high correlation between the HAAS-I and KOOS subscales (*r* = − 0.79 to *r* = − 0.91); and there were low correlations between the HAAS-I and SF-36 subscales (*r* = 0.01 to 0.29) for TKA.

**Conclusions:**

The HAAS-I was successfully translated into Italian and proved to have good psychometric properties that replicated the results of existing versions. Its use is recommended for clinical and research purposes.

## Background

The hip and the knee are two of the most common joints which develop primary osteoarthritis, which is mainly characterized by osteophyte formation, bone remodeling and subchondral sclerosis [1]. Osteoarthritis develops gradually over several years and, as it progresses, produces hip and knee dysfunction with reduced range of motion, muscle weakness and impaired proprioception, associated with limitations during walking, activities of daily living and working activities [2].

Orthopaedic surgery has rapidly developed over the last 20–30 years, and total hip and knee arthroplasty (THA and TKA, respectively) have now become the treatment of choice for subjects with intractable pain and severe disability from hip and knee OA. The number of THA in Italy increased 32% between 1999 and 2005, with about 50,000 new operations every year [3]. The number of subjects discharged after TKA in Italy significantly increased from 26,793 to 44,119 between 2001 and 2005, and surgical TKA revisions increased from 1166 to 2309 [3].

With such a high burden, it is of great importance to apply evidence-based, validated and comprehensive outcome measures to help clinicians to quantify and improve interventions. As supported by most researchers, a number of reliable and valid measures are available to assess functional outcomes following hip and knee arthroplasty, such as the Tegner and Lysholm score, the Oxford Knee Score (OKS), the Knee Society Clinical Rating System, the Harris Hip Score (HHS), the Merle d’Aubigne Hip score and the Western Ontario and McMaster Universities Osteoarthritis Index [4–8].

However, based on the good results of contemporary arthroplasty and the need to assess the performance in younger ages and high physical demands subjects, the High-Activity Arthroplasty Score (HAAS) was specifically developed to detect subtle variations in functional ability after lower limb arthroplasty [9]. It is a self-administered tool published in 2010 in English; it includes four domains (i.e. running, walking, stair climbing and overall activity), with each domain consisting of one item. Despite different levels of answers, a points system was applied to each option of each item, with a higher score indicating higher functional ability; the totals deriving from each domain are collected separately producing a total score, ranging from 0 (minimum) and 18 points (maximum in functional ability) [9].

To the authors’ knowledge, the HAAS has been cross-culturally adapted only in French [10]. However, non-English adaptations are of great interest as they contribute to investigate the psychometric properties of the original form of a scale, allowing comparison of results and investigating functional status across different people and countries.

### Purpose

A validation study of a translated form of the HAAS was never conducted in an Italian population. As this represented a limit for clinicians and researchers of our country to share validated outcomes, the aim of this study was to describe translation, cultural adaptation and validation (internal consistency, reproducibility, validity and sensitivity to change) of the Italian version of the HAAS in adult subjects after hip and knee arthroplasty.

## Methods

This cross-sectional study was approved by the Local Ethics Committee of the University of Cagliari and conducted in accordance with ethical and humane principles of research. Written consent to participate was obtained from all participants.

### Subjects

The study involved outpatients attending the Orthopaedics Unit at the Marino Hospital in Cagliari and the Physical Medicine and Rehabilitation Unit at the Tor Vergata Hospital in Rome between May 2016 and June 2017. The inclusion criteria were interventions for primary uncemented THA/TKA because of primary osteoarthritis 6 months before, adult age of < 65 years, and fluency in Italian; the exclusion criteria were cognitive impairment (i.e. Mini Mental State Examination of < 24) and neurological, heart and lung co-morbidities; subjects with previous lower limb surgery, infection, fracture, osteonecrosis or malignancy and systemic or neuromuscular diseases were also excluded.

Those subjects satisfying the inclusion criteria were asked to sign a written informed consent. Once the patients had given their approval to participate to the study, their demographic and clinical characteristics were recorded by research assistants.

### Cross-cultural adaptation

Adaptation of the HAAS was performed in accordance with the protocol issued by the American Association of Orthopaedic Surgeon Outcomes Committee [11]. Further, principles of good practice for the translation and cultural adaptation process for patient-reported outcomes (PRO) measures based on the report of the ISPOR task force were taken into account [12].

#### Step 1: translation into Italian

The items taken from the original scale were translated into Italian with the aim of retaining the concepts of the original while using culturally and clinically fitting expressions. Two translations were made independently by two Italian professional translators experienced in the PRO field. The translators were given a clear explanation of the concepts in the HAAS, to capture the conceptual meaning of the items. Keeping the language colloquial and compatible with a reading age of 12 years, discrepancies between the translators were resolved by means of reconciliation between them; step 1 ended when a common adaptation was agreed.

#### Step 2: back-translation into English

Two bilingual translators whose mother tongue was English independently back-translated the initial translation. The principal investigator (MM) reviewed these translations and, with the help of the back-translators, made sure that the Italian version reflected the same item content as the original version and was conceptually equivalent.

#### Step 3: expert committee

To harmonize the adaptation process, the translations were submitted to a bilingual committee of clinicians, methodologists and the translators, chaired by the principal investigator. To identify any discrepancies or mistakes, the committee explored the semantic, idiomatic and conceptual equivalence of the items and answers. This phase ended when a pre-final version was agreed.

#### Step 4: test of the pre-final version

This was performed to assess the level of understandability and cognitive equivalence of the translation, to highlight any items that may be inappropriate at a conceptual level and to identify any other issues that cause confusion. Cognitive interviews were therefore conducted by a trained psychologist by administering the HAAS to 10 patients with THA/TKA. The principal investigator and the Expert Committee reviewed the results from cognitive debriefing with the aim of identifying any modification necessary for improvement of the Italian form.

### Sample size

Sample size was based on the “rule of 10” patients per item [13].

### Scale properties

#### Feasibility

The time needed to answer the questionnaire was recorded. The subjects were asked about any problems they encountered, and the data were checked for missing or multiple responses.

#### Floor/ceiling effects

Descriptive statistics were calculated to identify floor/ceiling effects, which were considered to be present when > 15% of the subjects obtained the lowest or highest possible scores [13].

#### Reliability

Internal consistency (Cronbach’s alpha, with values of > 0.70 being considered acceptable) and test–retest reliability (intraclass correlation coefficient: ICC 2,1, with good and excellent reliability respectively indicated by values of 0.70–0.85 and > 0.85) [13] were investigated. The test–retest interval was 10 days.

#### Content validity

For the purpose of content validation, subjects were asked to report their perceptions of the aim of the measurement (question: “Do you think the aim of this questionnaire is to investigate high-intensity activities?”), the target population (“Do you think the items described here may be related to status?”), relevance (“Do you think these items are relevant to evaluating your high-intensity activities?”) and completeness (“Do you think that the items comprehensively reflect high-intensity activities?”). The hypotheses were considered acceptable if the percentage of affirmative answers was > 90% [13].

#### Construct validity

For construct validation [13], it was hypothesized a priori the HAAS would achieve moderate to high correlations with: (a) disability, the Italian version of Western Ontario and McMaster University index (WOMAC) for THA [14] and the Italian version of the Knee injury and Osteoarthritis Outcome Scale (KOOS) for TKA [15]; (b) pain intensity, the 0–10 Numerical Rating Scale (NRS) [16]; and (c) quality of life, the Italian version of the Short-Form Health Survey (SF-36) [17]. The correlations with these measures were expected to be moderate. Pearson’s correlations were interpreted as follows: *r* < 0.30 as low, 0.30 < *r* < 0.60 as moderate and *r* > 0.60 as high.

#### Sensitivity to change

It was estimated by means of the minimum detectable change (MDC) calculated by multiplying the standard error of the measurements (SEM) by the *z*-score associated with the desired level of confidence (95% in our case) and the square root of 2, which reflects the additional uncertainty introduced by using difference scores based on measurements made at two time points (in our case on days 1 and 10). The SEM was estimated using the formula: SEM = SD[(1 − *R*)^1/2^], where SD is the baseline standard deviation of the measurements and *R* the test–retest reliability coefficient [13].

### Measures

- *WOMAC*: It is a multidimensional scale of 24 items grouped into three subscales: physical function (17 items), pain (5 items) and stiffness (2 items); we used the 3.1 Likert version that allows five response levels for each item (scored 0–4) representing different degrees of intensity (none, mild, moderate, severe, or extreme). The data for each subscale are standardized to a range of 0–100, where 0 is the best and 100 the worst health status [14].
- *KOOS*: It has five subscales: Pain, Symptoms, Activities of Daily Living, Sport and Recreation and Knee-related Quality of Life. A 5-point Likert scale ranging from 0 (no problems) to 4 (extreme problems) is used to score each item, and the raw scores of each subscale are separately transformed into a 0–100 scale with 0 indicating the worst problems and 100 indicating no problems [15].
- *NRS:* This is an 11-point rating scale ranging from 0 (no pain at all) to 10 (the worst imaginable pain) [16]. Patients were asked to evaluate the pain they felt in the last week.
- *SF-36*: This is assessed using the Italian version of the self-report Short-Form Health Survey (SF-36). The eight domain scores (Physical Functioning, Physical Role, Physical Pain, General Health, Vitality, Social Activities, Emotional Role and Mental Health) were calculated on the basis of the Italian User’s Manual, with 0 representing the worst perceived QoL and 100 the best perceived QoL [17].

The analyses were made using the Italian version of SPSS 23.0 software.

## Results

### Subjects

The study involved 67 subjects with THA and 61 with TKA. There were 10 female (6.7%) patients who underwent a THA and 29 female patients who underwent a TKA (47.5%) with a mean age of 56.4 ± 6.8 years (range 43–65) for the first group and a mean age of 54.5 ± 6.1 years (range 45–64) for the second group. The median duration of complaints before intervention was 56 months (range 6–80) for THA and 53 months (range 6–48) for TKA. Their mean body mass index was 25.8 ± 3.2 for THA and 27.2 ± 5.4 for TKA. Table 1 shows socio-demographic characteristics.

**Table 1:** General characteristics of subjects with total hip arthroplasty (THA, *n* = 67) and total knee arthroplasty (TKA) (*n* = 61)

	THA		TKA	
Variable	*N*	%	*N*	%
Marital status				
Unmarried	17	25.4	12	19.7
Married	50	74.6	49	80.3
Employment				
Unemployed	13	19.4	4	6.6
Employee	21	31.3	27	44.3
Self-employed	1	1.5	4	6.6
Retired	21	31.3	14	23.0
Housewife	10	16.4	12	19.7
Education				
Elementary school	2	3.0	5	8.2
Middle school	28	41.8	26	42.6
Upper school	27	40.3	24	39.3
University	10	14.9	6	9.8
Smoking				
Yes	13	19.4	15	24.6
No	54	80.6	46	75.4
Use of drugs				
Antidepressants	5	7.5	7	11.5
Analgesics	15	22.4	15	24.6
Muscle relaxants	5	7.5	4	6.6
NSAIDs	7	10.4	3	4.9
None	35	52.2	32	52.5
Comorbidities (principal)				
Hypertension	15	22.4	12	19.7
Non-insulin dependent diabetes mellitus	2	3.0	2	3.3
Heart disease	4	6.0	5	8.2
Gastro-enteric disease	4	6.0	6	9.8
Respiratory disease	8	11.9	6	9.8
None	34	50.7	30	49.2

*NSAIDs* non-steroidal anti-inflammatory drugs

### Translation and cross-cultural adaptation

The translation procedure took 1 month to reach a culturally adapted version, and all the items were easily forward and back-translated; no difficulties were evidenced during the review of the back translations. The correctness of the process, the content of the items and the concepts expressed were confirmed by the experts. The cognitive interviews confirmed the comprehensibility and the cognitive equivalence of the translation; no other issues causing confusion were pointed out. Finally, the principal investigator and the Expert Committee confirmed the work performed.

The HAAS-I is reproduced in the Appendix.

### Psychometric scale properties

#### Acceptability

All of the questions were well accepted. The questionnaire was completed in 1.8 ± 1.3 min for THA and 1.6 ± 0.5 min for TKA. No missing responses or multiple answers were found. There were no problems in comprehension.

#### Reliability

Cronbach’s *α* was 0.85 for THA and 0.91 for TKA. Test–retest reliability was measured in all of the subjects and was excellent (ICC = 0.97 with 95% CI 0.95–0.98 for THA; ICC = 0.95 with 95% CI 0.92–0.98 for TKA).

#### Distribution and floor/ceiling effects

The adapted HAAS had no significant floor/ceiling effects in both populations (Tables 2 and 3).

**Table 2:** Distribution and floor/ceiling effects of HAAS-I and other measures in subjects with total hip arthroplasty

Outcome measures	Test mean (SD)	Re-test mean (SD)	Floor/ceiling effects (%)
HAAS	10.50 (3.62)	10.52 (3.42)	0/0 (0%,0%)
WOMAC	17.49 (22.40)	n.a.	10/0 (15%,0%)
WOMAC Pain	14.68 (20.81)	n.a.	21/0 (31%,0%)
WOMAC Stiffness	19.22 (25.41)	n.a.	32/0 (48%,0%)
WOMAC ADL	17.91 (22.91)	n.a.	11/0 (16%,0%)
NRS	2.01 (1.86)	n.a.	1/0 (2%,0%)
SF-36 Physical Function	67.23 (29.14)	n.a.	0/10 (0%,15%)
SF-36 Physical Role	46.30 (44.22)	n.a.	27/22 (40%,33%)
SF-36 Bodily Pain	62.06 (30.00)	n.a.	3/17 (5%,25%)
SF-36 General health	32.23 (16.90)	n.a.	0/0 (0%,0%)
SF-36 Vitality	59.86 (17.42)	n.a.	0/0 (0%,0%)
SF-36 Social function	72.38 (23.80)	n.a.	0/12 (0%,18%)
SF-36 Emotional Role	65.17 (45.48)	n.a.	20/40 (30%,60%)
SF-36 Mental Health	67.00 (21.26)	n.a.	0/4 (0%,6%)

*n.a.* not available, *SD* standard deviation, *ICC* intraclass correlation coefficient, *CI* confidence interval, *HAAS-I* High-Activity Arthroplasty Score (Italian version), *NRS* numerical rating scale, *WOMAC* Western Ontario and McMaster University index, *SF-36* Short Form Health Survey 36 items

**Table 3:** Distribution and floor/ceiling effects of HAAS-I and other measures in subjects with total knee arthroplasty

Outcome measures	Test mean (SD)	Re-test mean (SD)	Floor/ceiling effects (%)
HAAS	10.50 (3.62)	10.52 (3.42)	0/0 (0%,0%)
KOOS Symptoms	4.40 (3.60)	n.a.	10/0 (16%,0%)
KOOS Pain	4.72 (4.10)	n.a.	13/0 (21%,0%)
KOOS ADL	9.00 (8.50)	n.a.	15/0 (25%,0%)
KOOS Sport/Recreation	8.13 (5.03)	n.a.	0/0 (0%,0%)
KOOS Quality of Life	3.97 (2.54)	n.a.	6/0 (10%,0%)
NRS	1.90 (1.20)	n.a.	0/0 (0%,0%)
SF-36 Physical Function	78.20 (18.61)	n.a.	0/10 (0%,16%)
SF-36 Physical Role	76.23 (32.73)	n.a.	4/35 (7%,57%)
SF-36 Bodily Pain	80.57 (18.11)	n.a.	0/23 (0%,38%)
SF-36 General health	52.21 (10.18)	n.a.	0/0 (0%,0%)
SF-36 Vitality	69.67 (8.60)	n.a.	0/0 (0%,0%)
SF-36 Social function	84.01 (16.31)	n.a.	0/25 (0%,41%)
SF-36 Emotional Role	95.08 (21.80)	n.a.	3/58 (5%,95%)
SF-36 Mental Health	77.90 (9.70)	n.a.	0/0 (0%,0%)

*n.a.* not available, *SD* standard deviation, *ICC* intraclass correlation coefficient, *CI* confidence interval, *HAAS-I* High-Activity Arthroplasty Score (Italian version), *NRS* numerical rating scale, *KOOS* Knee injury and Osteoarthritis Outcome Scale, *SF-36* Short Form Health Survey 36 items

#### Content validity

The content of the items was considered valid for the evaluation of high-intensity activities. All the questions were judged relevant to investigate high-intensity activities in the populations investigated. The concepts being explored were clearly defined, and described high-intensity activities that might be influenced by THA and TKA.

#### Construct validity

Regarding the THA population, there was a moderate correlation between the HAAS-I and NRS (*r* = − 0.40), there was a high correlation between the HAAS-I and WOMAC (*r* = − 0.68) and there were moderate to high correlations between the HAAS-I and SF-36 domains (*r* = 0.34 to 0.63). Regarding the TKA population, there was a moderate correlation between the HAAS-I and NRS (*r* = − 0.77), there was a high correlation between the HAAS-I and KOOS subscales (*r* = − 0.79 to *r* = − 0.91) and there were low correlations between the HAAS-I and SF-36 domains (*r* = 0.01 to 0.29). Tables 4 and 5 summarize the correlations.

**Table 4:** Construct validity. Pearson’s correlations between the HAAS-I and WOMAC, NRS and SF-36 in subjects with total hip arthroplasty

Outcome measures	HAAS-I	*p* value
WOMAC	− 0.68	0.000
WOMAC Pain	− 0.66	0.000
WOMAC Stiffness	− 0.50	0.000
WOMAC Activities of Daily Living	− 0.70	0.000
NRS	− 0.40	0.001
SF-36 Physical Function	0.63	0.000
SF-36 Physical Role	0.46	0.000
SF-36 Bodily Pain	0.47	0.000
SF-36 General health	0.34	0.005
SF-36 Vitality	0.45	0.000
SF-36 Social function	0.42	0.000
SF-36 Emotional Role	0.48	0.000
SF-36 Mental Health	0.35	0.003

*HAAS-I* High-Activity Arthroplasty Score (Italian version), *NRS* numerical rating scale, *WOMAC* Western Ontario and McMaster University index, *SF-36* Short Form Health Survey 36 items

**Table 5:** Construct validity. Pearson’s correlations between the HAAS-I and KOOS, NRS and SF-36 in subjects with total knee arthroplasty

Outcome measures	HAAS-I	*p* value
KOOS Symptoms	− 0.80	0.000
KOOS Pain	− 0.82	0.000
KOOS ADL	− 0.89	0.000
KOOS Sport/Rec	− 0.91	0.000
KOOS QoL	− 0.83	0.000
NRS	− 0.76	0.000
SF-36 Physical Function	0.28	0.032
SF-36 Physical Role	0.29	0.024
SF-36 Bodily Pain	0.21	0.099
SF-36 General health	0.13	0.328
SF-36 Vitality	0.21	0.097
SF-36 Social function	0.06	0.636
SF-36 Emotional Role	0.26	0.026
SF-36 Mental Health	0.01	0.994

*HAAS-I* High-Activity Arthroplasty Score (Italian version), *NRS* numerical rating scale, *KOOS* Knee injury and Osteoarthritis Outcome Scale, *SF-36* Short Form Health Survey 36 items

#### Sensitivity to change

The MDC was 1.7 for THA and for 1.8 TKA, reflecting the smallest changes in score that are likely to reflect a true change rather than a measurement error.

## Discussion

This study describes the adaptation and validation of the HAAS-I in < 65 years old subjects who underwent primary uncemented THA/TKA because of primary osteoarthritis.

The results of the adaptation process indicate that it was successfully developed following international guidelines. The experts played an important role during the re-evaluation of the process and confirmed the quality of the work done. The on-field text confirmed that the items were easily understandable, leading to a valid measure of another culture’s concept of health that allows data comparability and cross-national studies.

The questionnaire was successfully self-administered and seems to be easily applicable in everyday clinical practice.

The HAAS-I was internally consistent, with estimated similar to original findings (THA/TKA: 0.86) and higher than French estimates (TKA: 0.58) [9, 10]. Test–retest reliability was also satisfactory; however, similar estimates were not calculated by the original developers as well as by French researches, and therefore, comparison cannot be conducted [10].

The HAAS-I had no serious floor/ceiling effects, demonstrating its ability to assess wider ranges of disease severity. No effects were also found in the French study for TKA [10].

Construct validity was initially analysed by comparing the HAAS-I with disability scales. The close correlations in both samples suggest that the theoretical constructs of the two measures are very similar. Significant correlations were found by the original developers concerning WOMAC (*p* < 0.001; Spearman coefficients not reported) and Harris Hip Score (*p* < 0.003; Spearman coefficients not reported), while non-significant estimates were for Oxford Knee Scale (*p* < 0.017; Spearman coefficients not reported) [9]; our findings were higher than French results when the HAAS was compared to Oxford Knee Scale (TKA: 0.19) [10]. The moderate to high associations with the NRS suggests that high intensity activities may be linked to the intensity of pain, particularly in subjects with TKA. The original and the French study did not investigate these relationships, and therefore, comparisons cannot be provided [10]. Construct validity was further analysed by relating the HAAS-I with quality of life. Moderate to high correlations were displayed for THA subjects, suggesting that the increase in high-intensity activities is correlated with an increase in perceived quality of life. Surprisingly, poorer correlations were found between the HAAS-I and SF-36 in TKA; the lower level of disability in our sample than previously reported [15] and the low rate of co-morbidities probably explain this unexpected result. Moreover, the distribution analysis showed that the SF-36 had floor and ceiling effects, which suggests uncertainty in the QoL answers. Again, comparison with other studies cannot be conducted.

HAAS-I proved to be also sensitive to change. Given the degree of repeatability, the SEM and SDC were reduced and ensured it could identify changes in the scores exceeding the threshold of instrument noise. At 95% confidence level, the minimum detectable change indicates that, if an individual shows a change of more than 1.7 points for THA and 1.8 points for TKA after a given intervention, it would not be a measurement error. In other studies, this estimate was never calculated and, therefore, comparisons cannot be made.

This study has some limitations. Firstly, as it was designed cross-sectionally, any significant correlations should not be confused with causal effects. Secondly, the relationships between high-intensity activities and physical tests were not considered because only questionnaires were used. Thirdly, content validity was based on questions that might have prevented neutral responses partially limiting the soundness of our results; the use of open questions in the future is suggested. Finally, a full psychometric assessment including also responsiveness was not conducted, and further studies are therefore recommended.

## Conclusions

The Italian version of the HAAS is reliable, valid and sensitive to change. It can be recommended for clinical and research purposes, and it is expected to improve the high-intensity activities assessment of < 65 years old subjects after hip and knee arthroplasty.

## Appendix

### High-Activity Arthroplasty Score (HAAS)—Italian version 1.0

Seleziona il più alto livello funzionale per ognuna delle seguenti categorie:

1. Camminare (max. 5 punti)

- 5 su terreni scoscesi e accidentati per più di un’ora
- 4 con difficoltà ma senza limiti su terreni accidentati pianeggianti
- 3 senza limiti su terreni non accidentati pianeggianti
- 2 su terreni pianeggianti per almeno mezz’ora
- 1 per brevi distanze senza assistenza (fino a 20 metri)
- 0 per brevi distanze o pochi passi, mediante ausili

2. Correre (max. 5 punti)

- 4 per più di 5 kilometri
- 3 blandamente per almeno 5 kilometri
- 2 attraversare di corsa la strada
- 1 fare qualche balzo per schivare il traffico, se necessario
- 0 impossibilitato

3. Salire le scale (max. 3 punti)

- 3 due scalini per volta
- 2 senza corrimano
- 1 usando corrimano o bastone
- 0 impossibilitato

4. Livello di attività (max. 6 punti)

- 6 sport competitivi (tennis in singolo, corsa per più di 10 kilometri, bicicletta per più di 80 kilometri)
- 5 sport amatoriali (tennis in doppio, sci, corsa per meno di 10 kilometri, sport aerobici ad alto impatto)
- 4 attività ricreative di alta intensità (passeggiata in collina, attività aerobica a basso impatto, giardinaggio, attività manuali, attività agricole)
- 3 attività ricreative di media intensità (golf, giardinaggio leggero, attività manuale leggera)
- 2 attività ricreative di bassa intensità (brevi camminate, bocce)
- 1 attività all’aperto solo se necessario (camminare per brevi distanze per fare shopping)
- 0 autonomia solo in ambito domestico

Punteggio totale:……….
